# A lasting crisis affects R&D decisions of smaller firms: the Greek experience

**DOI:** 10.1007/s10961-022-09957-7

**Published:** 2022-08-08

**Authors:** Ioannis Giotopoulos, Alexander S. Kritikos, Aggelos Tsakanikas

**Affiliations:** 1 Department of Economics, School of Economics and Technology, University of Peloponnese, Tripoli Campus, 22100 Tripoli, Greece; 2 German Institute for Economic Research (DIW Berlin), Berlin, Germany; 3 University of Potsdam, Potsdam, Germany; 4 GLO, Essen, and IAB, Nuremberg, Germany; 5 DIW Berlin, Mohrenstr. 58, 10117 Berlin, Germany; 6 Laboratory of Industrial and Energy Economics, School of Chemical Engineering, National Technical University of Athens, Zografou Campus, 15780 Athens, Greece

**Keywords:** Small firms, Large firms, R&D, Innovation, Productivity, Long-term crisis, L25, L60, O31, O33

## Abstract

We use the prolonged Greek crisis as a case study to understand how a lasting economic shock affects the innovation strategies of firms in economies with moderate innovation activities. Adopting the 3-stage CDM model, we explore the link between R&D, innovation, and productivity for different size groups of Greek manufacturing firms during the prolonged crisis. At the first stage, we find that the continuation of the crisis is harmful for the R&D engagement of smaller firms while it increased the willingness for R&D activities among the larger ones. At the second stage, among smaller firms the knowledge production remains unaffected by R&D investments, while among larger firms the R&D decision is positively correlated with the probability of producing innovation, albeit the relationship is weakened as the crisis continues. At the third stage, innovation output benefits only larger firms in terms of labor productivity, while the innovation-productivity nexus is insignificant for smaller firms during the lasting crisis.

## Introduction

Pursuing innovative strategies is critical for improving a firm’s output through increased productivity. What is already important in normal times (see *inter alia* Griffith et al., 2006, Hall et al., 2010; Huergo & Moreno, 2011; Baumann & Kritikos, 2016; Lööf et al., 2017) might become crucial in times of crisis: when firms are confronted with sharp reductions in sales, they must develop a sustainable and dynamic recovery path through an innovation strategy (Archibugi et al., 2013). However, when sales collapse, firms typically put R&D expenditures at the top of the list for cutting. Also public R&D investment often drops simultaneously in economies with moderate innovation activities; examples of this include Greece and other southern European economies (see Pellens et al., 2020). We use the 2008 Geek economic crisis to investigate how firms react in a situation of a deep and prolonged downturn. It is a fact that the 2008 global financial crisis created a turbulent environment for the Greek economy, being particularly harmful for the economic performance and viability of a large number of Greek firms (Giotopoulos et al., 2017; Williams & Vorley, 2015). In this paper, we analyze what kind of innovation strategies the Greek manufacturing sector, separated into small and large firms, practiced during a crisis that turned out not to be just a short shock but rather a long, unabated shock, as well as how the respective strategies influenced the firms’ productivity.

The long-term economic crisis in Greece offers a unique case study to explore how strong exogenous shocks to an economy affect small and large firms and their innovative behavior. Although the financial and debt crisis differs from the COVID-19 pandemic with respect to its sources, it shares two significant similarities. As Roper and Turner (2020) point out, both are sharp exogenous shocks rather than business-cycle fluctuations. Furthermore, both affected firms through strongly reduced liquidity, whether through a substantial reduction in the availability of commercial finance (economic crisis) or extensively reduced turnover (COVID-19 crisis) (see Fairlie, 2020, Fairlie & Fossen 2022). As the effects on business performance and the relevant business decisions may share common characteristics, an analysis could offer important insights for fine-tuning policy measures in the post-COVID era, especially in moderate innovation economies. In both cases, financial stringency will force firms to make rapid strategic decisions regarding spending and potential savings.

Using a unique Greek data set from a two-wave survey of 524 Greek manufacturing firms *during* the financial crisis period (2011 and 2013), we employ the well-established model of Crépon et al. (1998) to investigate how firms in different size groups of the manufacturing sector react as the crisis continued to trouble the economy. We find that, for the first stage of our analysis, the continuation of the crisis appears to be harmful for the R&D engagement of smaller firms while it increased the willingness for R&D activities among the larger ones. At the second stage, among smaller firms the knowledge production remains unaffected by R&D investments; among larger firms, the predicted R&D decision is positively correlated with the probability of producing innovation output. At the third stage, we observe that innovation output benefits only larger firms since it significantly improves their labor productivity.

With our analysis, we contribute to the literature on the effect of lasting economic shocks on R&D investments. We provide a systematic analysis on potential effect heterogeneities in R&D-innovation-productivity linkages for different firm size groups and we consider—to the best of our knowledge for the first time—how a *lasting* shock causes large economic imbalances in moderate innovator economies. This adds to the analysis on the effects of short-term shocks like the 2008 global financial crisis (as analyzed e.g. by Archibugi et al., 2013) as well as linkages between uncertainty and innovation of firms and the linkages between innovation activities and performance of SMEs for a large number of countries [as analyzed in two studies by Goel and Nelson (2021, 2022)]. In that sense, our research is relevant and novel as it may also allow for designing policy instruments intended to increase the resilience of firms across different size groups, i.e. small vs. large firms, through the R&D-innovation-productivity channel under prolonged turbulent economic conditions. Despite the fact that we analyze one country, we argue that our research has important implications for other countries as well. The unique empirical insights may be valuable for other moderate innovator economies that face such crises and are dominated by SMEs.

## Theoretical background, data and crisis measurement

### Theoretical background

Investments in R&D and innovation activities are, already in normal times, risky decisions aiming to increase the productivity performance of firms. To analyze this relationship, Griliches (1979) introduces a knowledge production function according to which investments into R&D increase the stock of knowledge, leading to innovation and, ultimately, to higher productivity. At the same time, such investments bear the risk of failure, as it might not be possible to realize positive returns on such investments (see *inter alia* Peters et al., 2017). The uncertainty from exogenous shocks may lead firms to delay or even abandon R&D projects, but uncertainty may also induce the introduction of cost-saving process innovations, thus acting as a hedge against risks (Goel & Nelson, 2021). There is also extensive research that empirically investigates—based on the Griliches (1979) knowledge production function and making use of the so called CDM model, a structural model introduced by Crépon et al. (1998)—the relationship between R&D, innovation, and labor productivity (see Hall, 2011, and Lööf, et al., 2017 for surveys).

Existing research also focuses on the question to what extent are smaller firms similarly able to manage R&D efforts to improve their stock of knowledge and to transfer this improved knowledge into higher productivity? Reasons for firm size differences are the two conditions driving this R&D decision: opportunity and appropriability (Cohen & Klepper, 1996). From related empirical research, we know that firm size is indeed positively associated with the decision to invest in R&D. However, smaller firms still substantially engage in R&D activities. The question driving this research is whether or not smaller firms benefit in a comparable way from innovation processes: do they increase their labor productivity in a way that is similar to large firms (see Hall et al., 2009; Baumann & Kritikos, 2016)? However, the impact of innovation activities on SME performance is a priori unclear, since process innovation may be cost-saving with respect to the production inputs or labor may exhibit strong complementarities with other inputs (Goel & Nelson, 2022).

In this contribution, we investigate how the triad relationship between innovation input, innovation output, and productivity develops during a prolonged economic crisis. When major exogenous shocks jeopardize markets, smaller businesses tend to be more vulnerable than their larger counterparts due to lack of resources, known as the liability of smallness (Eggers, 2020). In a lasting crisis, smaller firms may be reluctant, if not unable, to invest their limited resources into innovative projects with an uncertain outcome (Lee et al., 2015) or other activities that will increase their financial risks (Thorgren & Williams, 2020). This holds even more if firms will struggle to manage high levels of debt. Therefore, we aim to determine if smaller firms tend to refrain from investing in innovation activities during such long lasting crises.

### Data and crisis measurement

The data used to empirically investigate our main research question stem from an extensive field survey conducted through CATI method. The first wave took place in 2011, the second in 2013, with the same group of firms being surveyed. We should emphasize that both observation years refer to a crisis period that hit only the Greek economy particularly strong. The final sample used in this paper contains 524 Greek manufacturing firms that participated in both survey waves. Table 1 describes in detail the examined variables and presents per wave their frequency distributions for binary and 5-point Likert scale variables as well as some summary statistics for the continuous variables.

**Table 1:** Descriptive statistics of the examined variables

Variables	Description	2011 wave	2013 wave
In-house R&D	Firms indicated whether they have organized or developed an R&D department during the last two years	Frequency	
	1. Yes	25.96%	31.48%
	0. No	74.04%	68.52%
Innovation Output	Firms indicated whether they were engaged in new or significantly improved product or process innovations within the last two years	Frequency	
	1. Yes	67.37%	58.40%
	0. No	32.63%	41.60%
Training	Firms indicated whether they provided external or internal training programs to their employees within the last two years	Frequency	
	1. Yes	73.85%	72.85%
	0. No	26.15%	27.15%
Liquidity Constraints	Firms indicated (on a 1–5 Likert scale), the level of credit crunch conditions they face due to banks inability to provide loans	Frequency	
	1. None credit difficulties	25.96%	11.95%
	2. Low degree of credit difficulties	18.27%	9.06%
	3. Moderate degree of credit difficulties	19.42%	15.99%
	4. Relatively high degree of credit difficulties	17.12%	22.16%
	5. Very high degree of credit difficulties	19.23%	40.85%
Metropolitan	Firms indicated their location and based on this information a regional dummy was constructed referring to the two metropolitan areas of Greece, i.e. Athens and Thessaloniki	Frequency	
	1. The firm is located in the metropolitan areas of Greece	37.02%	
	0. The firm is located in the rest regions of Greece (i.e. non-metropolitan areas)	62.98%	
Labor Productivity	Sales per full time equivalent employees (in logs)	Summary Statistics	
	Mean	11.873	11.807
	Std Dev	0.833	0.925
	Max	14.685	15.174
	Min	6.463	7.875
Investment intensity	Capital investment per full time equivalent employees (in logs)	Summary Statistics	
	Mean	9.383	9.061
	Std Dev	1.435	1.313
	Max	15.807	13.074
	Min	3.912	4.855

We use the same set of firms, the firm size distribution of which is shown in Table 2, in both waves enabling thus to identify possible differences over time. As shown in Table 1, about 67% of the manufacturing firms^1^ of the sample have introduced a product or process innovation within the last two years of wave 1 (2011), whereas this rate falls to 58% in wave 2 (2013). About 25% of the sample indicated the existence of in-house R&D activities in 2011, which increased to 31% in 2013. Employee training is widely used, reaching 73% of the firms in both waves. Training costs seem to be unaffected and are not reduced despite the sharp increase in liquidity constraints. Liquidity constraints are substantial as the crisis continues and the percentage of firms that indicate a very high degree of bank credit difficulties, as it doubles between the two waves (from approximately 20% to 40%). Finally, the average values of labor productivity and capital investment remain almost stable in both waves (Table 1).

**Table 2:** Frequencies per Size Group

Size Group	Micro Firms (firms that employ fewer than 10 persons)	Small Firms (firms that employ 10–49 persons)	Medium Firms (firms that employ 50–249 persons)	Large Firms (firms that employ 250 or more persons)
% of firms	6.58%	40.94%	42.18%	10.31%

In this analysis, the crisis continuation variable is formulated with the value of 0 for the responses of 2011 and the value of 1 for the responses of 2013, the latter incorporating the peak of the Greek economic crisis.^2^ As a matter of fact, the recessionary cycle of the Greek economy began in 2008, along with the burst of the global economic crisis, when a first negative growth rate in the GDP was recorded (− 0.3%). By the end of 2011, the accumulative recession was − 18% of the Greek GDP, while at the end of 2013 Greece had lost 26.4% of its GDP. 2013 was the year when the Greek GDP was at its lowest level (measured in constant prices of 2015) since the outbreak of the crisis in 2008, thus representing the trough of the Greek experience. This is why we consider 2013 as a crucial milestone representing the worst moment of the Greek economic crisis (European Commission, 2017). The other important factor in this context is that over the following years (from 2014 onward) the Greek economy grew only slightly, if at all. Thus, after five years of strongly negative signs, the economy did not recover, rather it remained at a low level in economic stagnation before dropping by another 9% in 2020 in the wake of the pandemic. Overall the use of the crisis continuation dummy allows us to identify potential changes in firms’ innovation activities during a prolonged crisis, and especially in the Greek case as the crisis is deepening.

## Empirical strategy

To explore the relationship between a firm’s decision to invest in R&D, its innovation output and productivity, we apply the well-established three-stage CDM model (Crépon et al. 1998) by a variant developed by Mairesse et al. (2005). The general benefits of this framework are extensively described in various approaches (see Lööf et al., 2017), while the benefits of the variant by Mairesse et al. (2005) with respect to selection bias and endogeneity issues are discussed in Audretsch et al. (2020). The important difference of the model provided by Mairesse et al. (2005) is that it refers to the use of occurrence instead of intensity for R&D engagement and innovation. Hence, the selection bias for R&D intensity does not hold, for which Crépon et al. (1998) had to correct for in their specification. Thus, the Heckman selection approach is not necessary in the first stage of the CDM model when the variant of Mairesse et al. (2005) is applied. For the sake of brevity, we keep the model description short.

In the first stage, we use a bivariate probit model to estimate the innovation input; i.e., the probability of undertaking R&D activities (Mairesse et al., 2005). The decision of firm $i$ to invest in R&D at time $t$ $\left( {r_{i,t}^{*} } \right)$ can be specified as follows:

1 $$r_{i,t} = \left\{ {\begin{array}{*{20}l} {1,} \hfill & { if\quad r_{i,t}^{*} = {\rm X}_{i,t}^{{\prime }} a + D_{t} \rho + e_{i,t} > \hat{c}} \hfill \\ {0,} \hfill & {if\quad r_{i,t}^{*} = {\rm X}_{i,t}^{{\prime }} a + D_{t} \rho + e_{i,t} \le \hat{c}} \hfill \\ \end{array} } \right.$$

where $r_{i,t}$ represents the observed binary variable for the R&D decision, $r_{i,t}^{*}$ denotes an unobserved latent variable that captures the probability of undertaking R&D activities, ${\rm X}_{i,t}^{^{\prime}}$ is a vector of possible factors influencing the decision of firms to engage in R&D, and $e_{i,t}$ is the error term. When the unobserved latent variable exceeds a certain threshold level $\widehat{c}$, then the observed ${r}_{i,t}$ takes the value of 1, and 0 otherwise. ${D}_{t}$ denotes the crisis continuation dummy where in our analysis the first observation year (2011) takes the value of 0, while the second observation year (2013), where the crisis deepened, takes the value of 1.

In the second stage, the specification of the knowledge production focuses on the link between innovation input and innovation output. We use a probit model to estimate the probability of introducing an innovation output, where product and process innovation are merged to one variable of innovation output (Hall, 2011), by including the predicted R&D decision obtained from stage 1 as the explanatory variable. To this end, the knowledge production is modeled as:

2 $$i_{i,t} = \left\{ {\begin{array}{*{20}l} {1,} \hfill & { if\quad i_{i,t}^{*} = r_{i,t}^{*} \beta + {\rm Z}_{i,t}^{^{\prime}} \delta + D_{t} \lambda + u_{i,t} > \hat{c}} \hfill \\ {0,} \hfill & {if\quad i_{i,t}^{*} = r_{i,t}^{*} \beta + {\rm Z}_{i,t}^{^{\prime}} \delta + D_{t} \lambda + u_{i,t} \le \hat{c}} \hfill \\ \end{array} } \right.$$

where the observed binary variable for innovation output is denoted by $i_{i,t}$ and the latent R&D decision predicted in the first stage is represented by $r_{i,t}^{*}$. $Z_{i,t}^{^{\prime}}$ is a vector of factors that may influence the innovation output and $u_{i}$ is the error term.

The third stage of the CDM approach makes use of a productivity function including the predicted innovation output derived from stage two as the explanatory variable, as a proxy for knowledge input. To estimate the productivity, we use a Cobb–Douglas production function extended with the use of knowledge stock (Griliches, 1979). The equation of the OLS estimation is expressed in logs as follows:

3 $$y_{i,t} = \alpha_{1} + a_{2} k_{i,t} + a_{3} i_{i,t}^{*} + a_{4} W_{i,t} + D_{t} \mu + v_{i,t}$$

where the dependent variable $y_{i,t}$ denotes the labor productivity measured in sales per employees in logs. The explanatory variables of primary interest in the production function are the knowledge input ( $i_{i,t}^{*}$) derived from the estimated innovation output in stage 2, and the capital input ($k_{i,t}$) measured by the investment intensity in logs. Finally, $W_{i,t}$ is a vector of control variables, and $v_{i,t}$ is the observed error term.

## Results

### First stage: R&D engagement

We estimate the panel probit model expressed by Eq. (1) for the full sample and separately for the size groups,^3^ as defined above. Table 3 presents the marginal effects of the explanatory variables on the probability of firms’ engagement in R&D activities.

**Table 3:** R&D Engagement

Dependent Variable: In-house R&D	Full Sample	Micro and Small Firms < 50 employees	Medium and Large Firms > = 50 employees
50–249 employees	− 0.058		
	(0.048)		
10–49 employees	− 0.178***		
	(0.051)		
0–9 employees	− 0.440***		
	(0.095)		
Age class (15–34 years)	− 0.010	− 0.043	0.052
	(0.036)	(0.042)	(0.061)
Age class (35 + years)	0.010	− 0.012	0.037
	(0.042)	(0.055)	(0.065)
Exporting activity	0.027	0.033	0.041
	(0.037)	(0.044)	(0.059)
Metropolitan area	0.007	0.016	− 0.006
	(0.033)	(0.053)	(0.045)
Crisis Deepening	0.054	− 0.086**	0.199***
	(0.028)	(0.035)	(0.040)
Liquidity constraints	0.004	0.048	0.001
	(0.010)	(0.012)	(0.015)
Employees (ln)		0.073**	0.056**
		(0.031)	(0.025)
Industry dummies	Yes	Yes	Yes
Observations	1034	489	544

The table reports the marginal effects of the panel probit regressions

Reference groups for the firm size and age dummies: large firms (size group >  = 250 employees) and young firms (age group < 15 years)

The majority of the above variables are binary ones apart from Liquidity constraints (5-point Likert scale) and Employees (ln)

**Significance at *p* < .05 level; ***Significance at *p* < .01 level

Focusing on the total sample (Column 1), we find that micro and small firms are less likely to engage in R&D activities than the reference group^4^ of large-sized firms (confirming earlier findings of Hall et al., 2009 and Baumann & Kritikos, 2016).

To further explore whether the examined factors influence in a different way the R&D engagement of micro and small firms, as compared to their larger counterparts, we discuss the empirical results for the two size groups separately (Columns 2 and 3). In particular, the continuation of the crisis has a negative effect (significant at the 5% level) on the probability of micro and small firms engaging in R&D activities, while a positive and strong association (at the 1% level of significance) emerges in the case of larger firms. The coefficients’ values obtained from the marginal effects indicate that the continuation of the crisis is associated with an 8 pp decrease in the probability of smaller firms to become involve in R&D activities, while there is a 20% increase in the probability of larger firms to engage in R&D.

### Second stage: knowledge production

Table 4 presents the results from the second stage on the full sample and on the two examined size groups. For the full sample, we reveal a strong link between the predicted R&D (obtained from the previous stage) and innovation output in terms of probability (based on coefficients’ values) and significance level. Moreover, employee training is positively correlated with the probability of firms to innovate, while the continuation of the crisis and liquidity constraints are harmful for the firms’ innovativeness.

**Table 4:** Knowledge production function

Dependent Variable: Innovation Output (Product or Process Innovation)	Full Sample	Micro and Small Firms < 50 employees	Medium and Large Firms > = 50 employees
50–249 employees	0.325***		
	(0.478)		
10–49 employees	0.874**		
	(1.328)		
0–9 employees	2.248***		
	(3.315)		
Age class (15–34 years)	0.140**	0.223***	− 0.243**
	(0.222)	(0.283)	(0.501)
Age class (35 + years)	− 0.045	0.178	− 0.363***
	(0.218)	(0.320)	(0.539)
Training	0.127**	0.095	0.172**
	(0.204)	(0.282)	(0.340)
Investment intensity (in logs)	0.008	0.023	0.005
	(0.050)	(0.095)	(0.086)
In-house R&D (predicted)	1.630***	0.467	1.446***
	(2.241)	(1.432)	(2.254)
Metropolitan area	− 0.022	− 0.251	0.009
	(0.210)	(0.448)	(0.261)
Crisis Deepening	− 0.368***	0.182	− 0.964***
	(0.480)	(0.588)	(1.385)
Liquidity constraints	− 0.031**	− 0.017	0.005
	(0.057)	(0.084)	(0.076)
Employees (ln)		0.107	− 0.012
		(0.188)	(0.144)
Industry dummies	Yes	Yes	Yes
Observations	694	275	404

The table reports the marginal effects of the panel probit regressions

Reference groups for the firm size and age dummies: large firms (size group >  = 250 employees) and young firms (age group < 15 years)

**Significance at *p* < .05 level; ***Significance at *p* < .01 level. Bootstrap standard errors with 100 replications are reported in parentheses

Looking at the size groups, a strong link exists between R&D and innovation output for larger firms. Among smaller firms there is no such link. Additionally, training only unfolds a positive influence on innovation among larger firms, increasing the probability to innovate by 17 pp, while it has no effect on the innovativeness of smaller firms. However, the continuation of the crisis also decreases the probability of large firms to innovate by 9 pp. Last, but not least, among larger firms, it is particularly younger firms that are more likely to turn R&D into innovation output; among smaller firms this appears true at least for the middle-aged firms.

### Third stage: labor productivity

Table 5 presents the results for the third stage of the CDM model to reveal whether innovation activities affect the labor productivity of firms. Our findings for the full sample indicate that the productivity level of innovative firms is significantly higher compared to firms that do not innovate. Differentiating between firm size groups reveals that large firms are able to improve their labor productivity from innovation output, while the innovation-productivity nexus is insignificant for smaller firms. This finding raises similar concerns as those expressed in some empirical studies on manufacturing SMEs according to which product and process innovations may not necessarily foster firm productivity due to increased production costs associated with innovation investments (Jaumandreu & Mairesse, 2017; Exposito & Sanchis-Llopis, 2018).

**Table 5:** Production function

Dependent variable: labor productivity	Full sample	Micro and small firms < 50 employees	Medium and large firms > = 50 employees
Investment intensity (in logs)	0.198***	0.257***	0.179***
	(0.027)	(0.050)	(0.032)
Innovation Output (predicted)	0.311**	− 0.404	0.349***
	(0.129)	(0.216)	(0.106)
Employees		− 0.132	− 0.028
		(0.140)	(0.043)
Crisis deepening	0.125	0.008	0.156
	(0.075)	(0.122)	(0.084)
Industry dummies	Yes	Yes	Yes
Age group dummies	Yes	Yes	Yes
Size group dummies	Yes	No	No
Observations	557	207	350

Reference groups for the firm size and age dummies: large firms (size group >  = 250 employees) and young firms (age group < 15 years)

**Significance at *p* < .05 level; ***Significance at *p* < .01 level. Bootstrap standard errors with 100 replications are reported in parentheses

## Discussion and conclusion

We use the CDM model and data on 524 Greek manufacturing firms to explore how the prolonged Greek economic crisis that burst in late 2008 onwards affects the triad relationship between R&D, innovation output, and productivity. The most interesting result of the analysis is that important firm size differences emerge. For smaller firms an R&D decision has become less likely, while larger firms are even more likely to engage in R&D as the Greek crisis continued. These results imply different strategic responses when a crisis becomes long lasting between smaller and larger firms. Small and micro ventures might be constrained by a lack of resources due to the liabilities of smallness, where the continuation of the financial crisis might have led to a severe “funding gap” (Block & Sandner, 2009) resulting in reduced R&D expenditures and innovation efforts (Edeh & Acedo, 2021). By contrast, the crisis seems to have pushed large firms, which typically have better access to finance and other resources to withstand the economic downturn, to continue investing in R&D activities in order to create or further support a new competitive advantage either in the local market or in cross border markets (Geroski & Walters, 1995; Nickell et al., 2001). An additional aspect may be related to the fact that larger firms have already made some significant investments in R&D in either tangible or intangibles assets (see Le Mouel & Schiersch, 2020). Hence aborting such a plan maybe not an option for them due to sunk costs.

This has consequences for the later stages of our analysis: in the knowledge production function, a positive association between R&D and innovation output can only be found for larger firms, but not for smaller ones. Still, the continuation of the crisis also has negative effects for larger firms, as it reduces the probability of introducing an innovation among them. Hence, it seems that although larger firms are more likely to invest in R&D during turbulent economic times, diminishing returns may appear, taking the form of decreased innovation performance. These diminishing returns might be explained on the grounds of path dependencies of the past and of organizational inertia (Thrane et al., 2010), implying a limited agility of larger firms when significant changes emerge in the external environment, like those observed in adverse economic conditions.

Finally, innovation improves labor productivity only in larger firms, but the corresponding effect is not significant for smaller firms. The crucial link between R&D, innovation, and productivity that also exists for smaller firms in normal times (Baumann & Kritikos, 2016; Hall et al., 2009), is distorted during the continuation of the crisis, making them more vulnerable, and worsening their recovery from the shock (Castellani et al., 2019). When economic conditions worsen, smaller firms seem to reduce whatever R&D budget they had in place, affecting their innovative performance and their productivity. As a result of this, larger firms do have better survival probabilities during a long-lasting crisis, as they continue their innovation processes throughout such crisis times. This diverging result may lead to cleansing processes with smaller firms closing more often than larger ones—an outcome observed in the Greek manufacturing sector in subsequent years (see Kritikos et al., 2018).

The Greek governments had to follow specific adjustments programs, but also remained passive during these times, with the consequence that there was only a weak economic recovery. Economic stagnation persisted from 2014 through 2019 (resembling to an “L-shaped recovery”). This calls for a more active role at the policy level in order to overcome such a lasting crisis which is relevant in the context of the crisis following the COVID-19 pandemic. Our findings have useful implications not only for Greece but also for other moderate innovator economies. The structure of the economy in Greece with an overwhelming SME population is similar to several other EU countries with moderate innovation activities. Thus, our analysis offers unique empirical insights that can be valuable in such economies in designing policies to foster innovation and, consequently, the performance, competitiveness and resilience of SMEs during a lasting crisis caused by exogenous shocks.

In this context, we should emphasize that the economic shock due to the COVID-19 crisis is resembling in some parts to what Greece experienced during the 2008–2013 period in terms of some macroeconomic indicators. Both crises share common features, such as nearly double-digit GDP losses in economies with moderate innovation activities, increasing unemployment rates, negative inflation rates, as well as significant increases in public debt (European Commission, 2020). In both cases, the regulatory frameworks, institutions, and investors were unprepared for the magnitude and the persistent consequences of the crises (Lustig & Mariscal, 2020). Both crises also appear to have devastating effects on business activity, resulting in business exits, supply chain disruptions, redundancies, and loss of key customers (Belitski et al., 2022). And there are visible signs for diverging funding pattern at the first stage of the relationship between R&D, innovation and productivity: smaller firms reduced their R&D investments during the first year of the COVID-19 crisis (Infas et al. 2021).

In contrast to the rather passive role of the Greek governments and the fiscal consolidation process underway that restricted interventions during the Greek economic crisis, under the COVID-19 crisis funds from the EU’s Resiliency and Recovery Facility (RRF) were quickly available, aiming to mitigate the economic and social impact of the pandemic crisis. Basically, there are two options that may facilitate a quick recovery when making use of these funds. One option could be to secure additional support for small and micro firms during such turbulent times. Measures like extensive tax reductions on R&D expenses, over-depreciation rates on R&D equipment, an increase in public R&D funding, and swifter regulation for attracting researchers to contract-based research or collaborations with universities (Fernandes & O’Sullivan, 2022) could offer additional incentives for overcoming “R&D crunch” conditions among smaller businesses. Any type of support in collaboration in R&D for smaller firms is beneficiary (Matt et al., 2012) as these firms rely more on external sources of input to the innovation process and as smaller firms tend to receive greater benefit from such exchange. Overall, such a policy mix would support smaller firms (Petrin & Radicic, 2021) to survive such long-lasting crisis and secure growth prospects afterwards. The other option could be to simply let some less efficient small and micro firms exit the market, which could create an opportunity to increase the notoriously underrepresented number of large firms in such moderate innovation economies by removing regulatory obstacles that hinder productivity (Kilinç 2018) and the growth of the remaining smaller firms (Herrmann & Kritikos, 2013), thus increasing labor productivity by supporting transitions from small to large firms.

## Footnotes

1 Note that our empirical work focuses on the manufacturing sector. The Oslo Manual and several studies emphasize that fundamental differences in the innovation process exist between manufacturing and the service sectors (Audretsch et al., 2020; Becheikh et al., 2006; Ettlie and Rosenthal 2011).

2 In this context, we need to emphasize one limitation of our study. There are no sufficient data on the Greek manufacturing sector from the previous non-crisis period (prior to 2008) that do allow to make a comparison to these years. The Greek data from the (typically used) community innovation survey (CIS) miss information on the labor force so that it is not possible to estimate effects on labor productivity.

3 Following Baumann and Kritikos (2016) we split the sample to provide separate estimations per size group (small versus large firms), since this enables the exploration of heterogenous effects between small and large firms.

4 As a common practice in estimations with k group dummies, we include k-1 variables to avoid perfect multicollinearity and the missing group is considered as the reference group in the interpretation of the estimated coefficients of the k-1 dummies (in our case the group dummies refer to size and age groups). In reporting the estimation results from the probit regressions in the first two stages we use marginal effects and the results referring to the missing reference group are discussed implicitly. The constant term and subsequently more direct results for the missing dummy group can be extracted from the initial probit estimations, i.e. before the computation of the marginal effects. These results are not reported since they are not of interest given the scope of the current study; however, they are available from the authors upon request.
